# Factors associated with aspects of functioning one year after hospitalization due to COVID-19

**DOI:** 10.1177/02692155241311852

**Published:** 2025-01-07

**Authors:** Alexandra C. Larsson, Annie Palstam, Linda Ashman Kröönström, Katharina S. Sunnerhagen, Hanna C. Persson

**Affiliations:** 1 Department of Clinical Neuroscience, Institute of Neuroscience and Physiology, Sahlgrenska Academy, University of Gothenburg, Gothenburg, Sweden; 2 Department of Occupational Therapy and Physical Therapy, 56749 Sahlgrenska University Hospital, Gothenburg, Sweden; 3 Department of Rehabilitation Medicine, 56749 Sahlgrenska University Hospital, Gothenburg, Sweden; 4 School of Health and Welfare, Dalarna University, Falun, Sweden

**Keywords:** COVID-19, functioning, functional outcomes, rehabilitation, hospitals, respiration, activity, body function, post-COVID

## Abstract

**Objective:**

To identify factors, present at 3 months after COVID-19 that are associated with the level of functioning 1 year after hospitalization.

**Design:**

Multicenter prospective observational study.

**Setting:**

Region Västra Götaland Sweden.

**Participants:**

Patients ≥ 18 years of age who were followed regarding body functions and activities 3 months and 1 year after discharge from a hospitalization for COVID-19.

**Main measures:**

Patient-reported outcome measures at 3 months and 1 year, a clinical follow-up at 1 year, and clinical information retrieved from medical charts.

**Results:**

In total 169 participants were included in the analysis, including 113 males (67%). The mean patient age was 66 (standard deviation (SD) 13, range 21–95). One year after hospitalization, 50 (34%) participants were under the normative value for forced vital capacity and 57 (39%) were under normative value for forced expiratory volume in 1 second. The mean walking distance was 441 m (SD 118 m) in the 6-minute walking test, and 62 (40%) patients reported mobility problems. Older age, female sex, and more self-reported symptoms of physical fatigue were significantly associated with lower levels of functioning 1 year after COVID-19. The initial severity of COVID-19 did not significantly influence functioning at 1 year.

**Conclusion:**

Previously hospitalized individuals with respiratory difficulties 1 year after COVID-19 may present acceptable lung function on spirometry but be close to their maximal functional performance. The findings highlight the need for in-depth assessments to identify rehabilitation needs.

## Introduction

Post-COVID syndrome is defined as symptoms lasting for at least 3 months after the onset of infection that cannot be explained by other illnesses.^1^ These symptoms often limit everyday activities and diminish the quality of life for those affected.^1^ Reduced functioning and impaired quality of life have been seen in individuals who were hospitalized because of COVID-19.^2^ Similarly, persisting impairment of functional status and low quality of life is still common in previously infected individuals.^2^

Studies highlight that individuals hospitalized for COVID-19 experience more symptoms and show a slower decline in symptom severity over time, compared to less severe cases who did not require hospitalization.^2–4^ The International Classification of Functioning, Disability and Health (ICF) framework illustrates the complexity of health and disability in relation to specific health conditions, such as COVID-19 or post-COVID syndrome, by using the ICF term “functioning” to describe an individual's body functions, activities, and participation.^5,6^ Some commonly occurring functional impairments after hospitalization for COVID-19 are activity limitations and impairments in body functions, such as pain and symptoms of anxiety and depression.^2^ Furthermore, body functions, such as pulmonary capacity and exercise capacity, seem to improve over time, in contrast to fatigue, which may have a delayed onset and increase over time.^7^ Being more physically active is associated with feeling less fatigue, even when adjusted for age, sex, and socioeconomic factors.^8^ Not being able to engage in physical activity due to fatigue after COVID-19 has been seen to affect quality of life and functional status.^9^

Although increased levels of physical activity may improve quality of life in individuals recovering from COVID-19, the factors early in the rehabilitation process that are associated with lower levels of functioning 1 year after hospitalization due to COVID-19 have yet to be determined. Countries adopted different strategies in managing the pandemic, where Sweden used less intense government restrictions.^10^ The different approaches could have influenced the patterns of infection, hospitalization and recovery and the long-term impact on functioning due to COVID-19 has not been studied extensively in a Swedish context.

The aim of this study was to identify factors present 3 months after hospitalization due to COVID-19 that are associated with the level of functioning 1 year later.

## Methods

### Design and participants

*Life in the Time of COVID Study in Gothenburg* is a multicenter prospective observational study. The study enrolled participants hospitalized with COVID-19 throughout the Region Västra Götaland between July 2020 and February 2021, the duration of the first and second waves of the pandemic and prior to the COVID vaccine being available to the public in Sweden. Two follow-ups were conducted: the 3-month follow-up took place between September 2020 and June 2021, followed by the 1-year follow-up, which was conducted between July 2021 and March 2022.

Patients were included if they were  ≥ 18 years old, had previously lived independently, had a hospital stay for ≥5 days, and were not contagious when enrolled. Patients were excluded if they were not able to provide informed consent or had prior severe illness with high 1-year mortality according to the responsible physician. Patients who were not Swedish residents were also excluded due to not being able to partake in the study follow up. All participants signed informed consent prior to enrollment in the study.

The study was approved by the Swedish Ethical Review Authority (Dnr: 2020-03046, 2020-03922, 2021-00444, 2021-03556) and complies with the Declaration of Helsinki. Two patient partners were involved throughout the project, one man previously admitted for COVID-19 and a woman who was treated at home. The STrengthening the Reporting of OBservational studies in Epidemiology guidelines (STROBE) were followed when drafting this manuscript.^11^

### Data collection

Participants were contacted via telephone and sent patient-reported outcome measures by mail both 3 months after hospital discharge and prior to the 1-year follow-up. The 3-month follow-up consisted of a telephone interview based on the patient reported outcome measures. At 1 year, the participants were assessed at a clinical follow-up (Table 1). For those unable to attend in-hospital follow-up, the follow-up was conducted in the participant's home. The clinical visits, scheduled for approximately 60 minutes, were performed by a physiotherapist trained within the project. Participants who were unreachable were sent a letter with a proposed time for a clinical visit.

**Table 1.** Self-reported functioning, assessment of functioning, and follow-up time points.

Patient-reported outcome measures	Acute care setting	3-Month follow-up	1-Year follow-up
CAT		X	X
HADS		X	X
MFI-20		X	X
PCFS	X	X	X
SGPALS	X	X	X
EQ5D-3L		X	X
Assessments			
Spirometry			X
Heartrate at rest	X		X
SpO_2_ at rest	X		X
30sCST	X		X
JAMAR	X		X
6MWT			X

6MWT, 6-minute walking test; 30sCST, 30-second chair stand test; CAT: chronic obstructive respiratory disease assessment test; EQ5D-3L: EuroQol-5Dimension3Levels; HADS: Hospital Anxiety and Depression Scale; JAMAR, hand dynamometer strength test; MFI-20: multidimensional fatigue inventory; PCFS: post-COVID functional status; SGPALS: Saltin Grimby physical activity level scale; SpO_2_, peripheral capillary oxygen saturation; .

The present study included data from the 3-month telephone follow-up and the 1-year clinical follow-up and additional clinical information retrieved from medical charts at hospital discharge for COVID-19 (Table 1). Clinical characteristics included level of care (intensive care or regular medical ward), level of respiratory support (intubation or noninvasive ventilation support), and length of hospital stay. Comorbidities were categorized using the Charlson Comorbidity Index as: no comorbidities (0 points), mild (1–2 points), or severe (>2 points).^12,13^ COVID-19 severity was categorized using the World Health Organization Clinical Progression Scale, dichotomized as “moderate” or “severe.” Moderate infection required hospital care with or without oxygen therapy (score 4–5), while severe infection required advanced respiratory or medical support, such as noninvasive ventilation, mechanical ventilation, or vasopressors (scores 6–9). Not all participants with severe COVID-19 required ICU admission, as advanced respiratory support (such as noninvasive ventilation) also was provided in specialized wards or infection clinics, with ICU admission typically reserved for intubation or scores ≥7.^14^

Level of functioning 1 year after hospitalization due to COVID-19 was explored using the *International Classification of Functioning* domains: body functions and activities.^5,6^ Self-reported or assessed body functions included respiratory, cardiovascular, mental, and muscle functions while activities encompassed self-care and domestic life recreational and leisure activities. Contextual factors, such as age, sex, and COVID severity, were noted. Additionally, participants self-reported health-related quality of life.

### Self-reported level of functioning and quality of life

Physical activity after COVID-19 was self-reported using the *Saltin-Grimby physical activity level scale*, scored as 1 (physically inactive), 2 (some light physical activity > 4 hours/week), 3 (regular physical activity and training > 2–4 hours/week), or 4 (regular hard physical training for competitive sports several times/week).^15^ Respiratory symptoms were self-reported using a modified version of the *chronic obstructive pulmonary disease assessment test*,^16^ adapted for COVID-19.^17^ It includes eight Likert scale questions (0 = no to 5 = severe impairments; maximum total score of 40). Previous lung disease could also be reported.^16^ Symptoms of anxiety and depression were assessed using the *Hospital Anxiety and Depression Scale*, which ranges from 0 to 21 points per domain.^18^ Perceived fatigue was assessed using the *Multidimensional Fatigue Inventory*, covering 5 domains (mental fatigue, reduced motivation, reduced activity, general fatigue, and physical fatigue) scored from 4 to 20 per domain, with higher scores indicating more fatigue.^19^

At the 1-year follow-up, participants reported their health-related quality of life using the 3-level version of the *EuroQol-5 Dimensions *^20^ scale, which assesses 5 dimensions (mobility, self-care, activity, pain, and anxiety/depression). Each dimension is rated on a three-point Likert scale (1 = mild problems, 3 = extreme problems). The outcome measure also includes a visual analog scale (0–100) for perceived health status, with 100 indicating the best possible health. Self-reported *post-COVID functional status *^21^ was also assessed at the 1-year follow-up. Participants rated their perceived functional limitations, from “no functional limitations” (0 points) to “severe functional limitations” (4 points).

### Assessed level of functioning

Respiratory function was evaluated by spirometry using a portable spirometer (EasyOne Air, Medical Technologies, Switzerland). Participants received instructions on how to perform the test, which was conducted following the European Respiratory Society standards.^22^ Each participant performed three attempts, in a standardized manner, and the mean value was calculated. Spirometry data were analyzed as the percentage of a predicted reference value based on height, age, and sex according to spirometry standards^23^ with a normative value defined as the lower limit of normal (≥80% of the predicted value).^23^ Dynamic lung volumes that were measured included forced vital capacity, forced expiratory volume in 1 second, and peak expiratory flow.

Muscle strength in the lower extremities was assessed using the *30-second chair stand test*.^24^ Participants were instructed to rise from a seated position on a chair with a backrest, whit their arms crossed at the chest, as many times as possible for 30 seconds. The total number of repetitions was noted. Saturation and heart rate were monitored at the start and end of the test using pulse oximetry with a finger probe (Nonin onyx vantage 9590, Nonin Medical, Inc., Minnesota, USA). Results were compared with normative data from older adults.^25^

Muscle strength in the upper extremities was determined by the hand muscle strength, which was assessed using a JAMAR hand dynamometer (Sammson Preston, Chicago) in a standardized manner.^26^ Three attempts were made for each hand, and the mean values were calculated and presented as a descriptive of functioning 1 year after COVID-19. The values were also compared to reference values.^27^

The *6-minute walk test* was used to submaximaly assess walking capacity according to guidelines from the American Thoracic Society.^28^ Participants were instructed to shuttle walk a 30-meter hallway continuously for 6 minutes. If the participant stopped before the 6 minutes had passed, the number of meters walked before stopping was noted. Ratings of perceived exertion were assessed using the Borg scale and the category scale with ratio properties for perceived dyspnea.^29^ Saturation and heart rate were monitored by pulse oximetry using a finger probe (Nonin onyx vantage 9590, Nonin Medical, Inc., Minnesota, USA). Desaturation was defined as saturation  < 90% directly after the test.^30^

### Statistical analysis

All analyses were performed using IBM Statistical Package for Social Sciences (SPSS) 27 with statistical significance set at *p* < 0.05. Participant characteristics are presented as frequencies (*n*) and percentages (%), mean with standard deviations (SD), medians with ranges (minimum–maximum), or inter quartile ranges (IQR). If missing in one single item in the *chronic obstructive pulmonary disease assessment test* imputation according to the “half-rule” (adding the mean score of all the items combined into the missing item) was performed,^31^ used in four participants. Multivariable linear regression analysis was performed to analyze factors at 3 months that were potentially associated with functioning 1 year after COVID-19, with walking distance (*6-minute walk test*) as the dependent variable and defined as a proxy for functioning 1 year after hospitalization due to COVID-19. Independent variables at 3 months included in the model were physical activity level, respiratory symptoms, symptoms of anxiety and depression, level of fatigue, COVID-19 severity, age, and sex. Independent variables were tested for multicollinearity using Spearman correlation (Rho > 0.7). Multicollinearity was present in the *Hospital Anxiety and Depression scale*, and the depression domain was included in the model. Multicollinearity was also present in the physical fatigue and general fatigue domains of the *Multidimensional Fatigue Inventory scale*, and the physical fatigue domain was included in the model. Initial analysis of assumptions regarding normality, outliers, and linearity was conducted using scatterplots for continuous data and boxplots for categorical data. The final model included age, sex, COVID severity, level of physical activity, level of respiratory symptoms, level of depressive symptoms, and level of physical fatigue. Sensitivity analyses were carried out excluding outliers. One case was excluded in the model due to severe impairments unrelated to COVID-19. Standardized confidence intervals were calculated using Microsoft Excel (version 2024) SE*=β*/β , where SE* indicates standardized standard error, β* standardized beta coefficients (regression coefficient), and *β* unstandardized beta coefficient. The explained variance in the dependent variable was described by the coefficient of determination, *r*^2^. Figures were created using GraphPad Software (version 2024).

## Results

**Figure 1.**
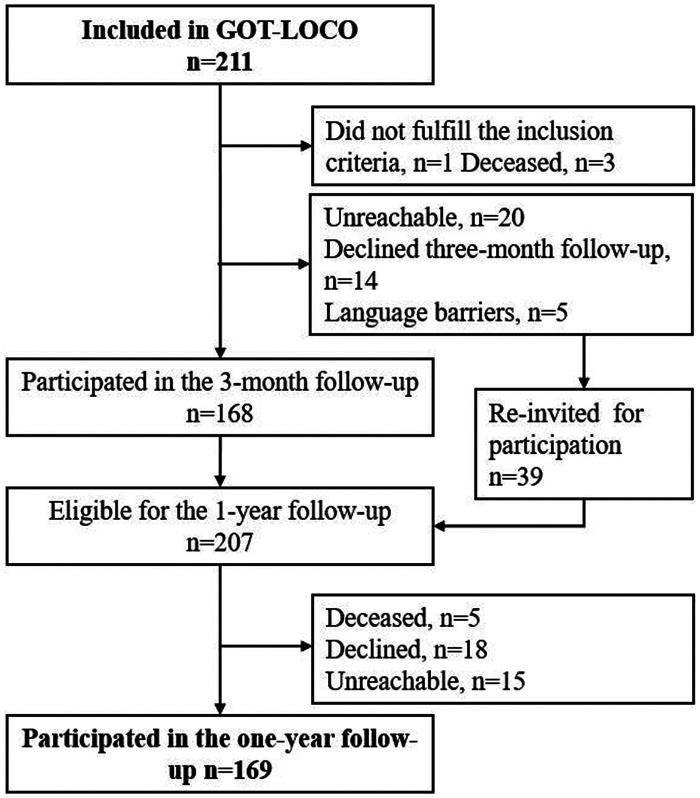
Flowchart of inclusion, participants from life in the time of COVID study in Gothenburg.

Of the 211 participants included in *Life in the Time of COVID Study in Gothenburg* one was excluded due to the exclusion criteria and 8 died before the 1-year follow-up. Thus, a total of 202 participants were invited to the 1-year follow-up: 169 (80%) individuals with a mean age of 66 years (SD 13) participated in the follow-up and were included in the present study (Figure 1).

Of the included participants, 113 (67%) were male. The majority of participants (67%) had severe COVID-19 infection. The participants were hospitalized for a median 18 days (5–195 days; Table 2).

**Table 2.** Participant characteristics (*n* = 169).

Median age, years, n (min–max) Age groups, ≥65 years /<65 years, *n* (%)	67 (21–95) 90 (53)/79 (47)
Sex: male/female, *n* (%)	113 (67)/56 (33)
**Clinical characteristics at hospital admission**	
Length of hospital stay, days mean (SD)	33 (37)
median (min–max)	18 (5–195)
ICU admitted, *n*, (%)	88 (52)
–Intubated *n* = 156	56 (36)
NIV *n* = 163 *n*, (%)	104 (62)
COVID-19 severity, *n*, (%)	
–Moderate	56 (33)
–Severe	113 (67)
CCI at hospital discharge, *n*, (%)	
–No comorbidities (0 comorbidities)	66 (32)
–Mild comorbidities (1–2 comorbidities)	95 (46)
–Severe comorbidities (>2 comorbidities)	48 (22)
Education *n* = 157, *n* (%)	
–Elementary school, ≤9 years	33 (21)
–High school:10–12 years	82 (52)
–University: >12 years	42 (27)
**Characteristics at the 1-year follow up**	
**Work status** *n* = 145, *n*, (%)	
–Retired	87 (60)
–Working	50 (35)
–Unemployed	4 (3)
–Sick leave	2 (1)
–Studying	2 (1)

Values are presented as n and percentage (%) unless otherwise noted.

CCI: Charlson Comorbidity Index; ICU: intensive care unit; max: maximum; min: minimum; NIV: noninvasive mechanical ventilation; SD: standard deviation; .^12^

### Functioning

**Figure 2.**
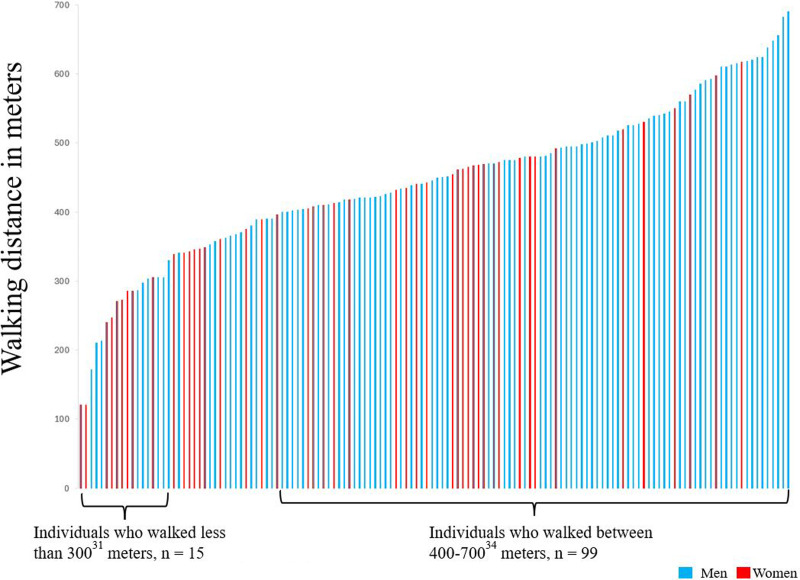
Results of walking distance.

At 1 year, 4% of participants had hand muscle strength above normative values for the right hand based on age and sex. Higher levels of strength were also observed in the left hand, 17% were above reference values (Table 3). Regarding muscle strength in the lower extremities, the mean number of chair rises was 11.8 (SD 5.3). The mean distance walked for the whole group was 441 m (SD 118 m) and there were 13 participants (11%) who walked ≤300 m (Figure 2). After completing the *6-minute walk* test, the median ratings of perceived exertion were 13 (“somewhat hard”) and the dyspnea category scale was 3 (“moderate”). Fifteen percent of participants had saturation < 90% after the test. Regarding respiratory function, 34% (*n* = 50) were under the normal values (<80% lower limit of normal) for forced vital capacity and 39% (*n* = 57) were under the normal values for forced expiratory volume in 1 second. Of the participants who were under the lower limit of normal for forced expiratory volume in 1 second, 14 (9%) reported pulmonary disease (chronic obstructive pulmonary disease, *n* = 10, asthma, *n* = 3, and sarcoidosis, *n* = 1).

**Table 3.** Assessed level of functioning 1 year after hospitalization due to COVID-19.

		Participants	Under 65 years	Over 65 years	Men	Women
		Mean ± SD	Mean ± SD	Mean ± SD	Mean ± SD	Mean ± SD
Walking distance						
	6MWT *n* = 140, m	441 ± 118	475 ± 119	405 ± 107	465 ± 110	390 ± 122
	SpO_2_% before 6MWT	97 ± 1.9	96.8 ± 1.5	96.6 ± 2.3	96.6 ± 2.1	97.2 ± 1.5
	SpO_2_% after 6MWT	93.5 ± 4.5	93.8 ± 4	93 ± 4.6	93 ± 4	93.7 ± 4.9
Respiratory function						
	Spirometry *n* = 148 FVC, l/m	3.2 ± 1	3.6 ± 0.9	2.9 ± 0.9	3.6 ± 0.9	2.6 ± 0.9
	FEV1, l/s	2.5 ± 0.8	2.9 ± 0.8	2.1 ± 0.8	2.8 ± 0.8	1.9 ± 0.8
	PEF, l/m	6.3 ± 2.8	7.4 ± 2.7	5.3 ± 2.5	7 ± 2.8	5 ± 2.3
Muscle function	Leg strength *n* = 158 30sCST	11.8 ± 5.3	13 ± 5	10 ± 5	12 ± 6	11 ± 5
	SpO_2_% before 30sCST	96 ± 2.5	96.3 ± 2.3	95.5 ± 2.8	95.6 ± 2.4	96.4 ± 3
	SpO_2_% after 30sCST	95 ± 3.5	95.4 ± 4	94.8 ± 3	95 ± 3.6	95.2 ± 3.6
	Grip strength, JAMAR, kilos					
	Right hand *n* = 163 *percentage of predicted results*	36.5 ± 13.7 104 ± 28.6	40.6 ± 14.8 100.7 ± 29.9	32.8 ± 11.5 108.6 ± 27.1	42.4 ± 12 106.3 ± 27.8	24.6 ± 8.1 102 ± 30.3
	Left hand *n* = 162 *percentage of predicted results*	35 ± 14 117 ± 38	38.9 ± 15.4 111 ± 36.2	31.3 ± 11.9 122.9 ± 38.2	40.8 ± 12.9 120. ± 37.7	23.5 ± 8.5 110.9 ± 37.1

Data is presented as mean and standard deviation (±) unless specified as other. The variation in the number of participants in each test are mainly due to missing values depending on poor physical functioning or inability to standardize test procedure due to home visits.

6MWT: 6-minute walk test; 30sCST: 30-second chair stand test; FEV_1_: forced expiratory volume in one second; FVC: forced vital capacity; JAMAR: hand grip dynamometer; PEF: peak expiratory flow; SD: standard deviation; SpO_2_: peripheral capillary oxygen saturation.

### PROMs 1 year after hospitalization due to COVID-19

The self-reported respiratory symptoms (median 14) points indicate persisting respiratory symptoms 1 year after hospitalization for COVID-19 (Table 4). Nine percent (*n* = 16) of participants reported severe functional limitations 1 year after COVID-19, in contrast to the 72% (*n* = 119) who reported no, negligible, or slight *post COVID functional status* limitations (Table 4). Of the participants, 53% (*n* = 88) graded their physical activity level as light, indicating at least 4 hours of physical activity a week. Regarding health-related quality of life 52% (*n* = 80) reported moderate pain and 10% (*n* = 15) extreme pain, indicating that approximately two-thirds of the cohort reported general pain 1 year after hospitalization due to COVID-19.

**Table 4.** Patient-reported outcome measures.

EuroQol 5 dimensions *n* = 154	Possible range 1–3	Specific domain or grade of problems	*n* (%)
Mobility		1, Mild 2, Moderate 3, Severe	92 (60) 62 (40) —
Self-care		1, Mild 2, Moderate 3, Severe	141 (92) 13 (8) —
Activity		1, Mild 2, Moderate 3, Severe	113 (73) 35 (23) 6 (4)
Pain		1, Mild 2, Moderate 3, Severe	59 (38) 80 (52) 15 (10)
Anxiety/depression		1, Mild 2, Moderate 3, Severe	93 (60) 55 (36) 6 (4)
EuroQol 5 dimensions *n* = 140 median (min–max) mean ± SD		Visual analog scale	70 (15–100) 66 ± 21
COPD assessment test *n* = 154	0–40		Median [IQR]
			14 [8,21]
Hospital anxiety and depression scale *n* = 154	0–21		Median [IQR]
		Anxiety depression	4 [2,8] 4 [1,7]
Multidimensional fatigue inventory *n* = 154			Median [IQR]
	4–20 4–20 4–20 4–20 4–20	General fatigue *n* = 148 Physical fatigue *n* = 152 Mental fatigue *n* = 151 Reduced activity *n* = 151 Reduced motivation *n* = 151	14 [11,16] 13 [11,17] 10 [7,14] 13 [10,16] 10 [7,12]
Post-COVID functional status *n* = 153	0–4		*n* (%)
		0, No functional limitations 1, Negligible functional limitations 2, Slight functional limitations 3, Moderate functional limitations 4, Severe functional limitations	47 (28) 28 (17) 44 (27) 31 (19)
Saltin Grimby physical activity level scale *n* = 153	1–4		*n* (%)
		1, Physically inactive 2, Light physical activity 3, Moderate physical activity 4, Vigorous physical activity	52 (31) 88 (53) 25 (15) 2 (1)

Data are presented as *n* (%) or median and inter quartile range [IQR]: PROMs, patient-reported outcome measures. Missing data was due mainly to participants not filling out single items.

CAT: COPD assessment test; EQ5D-3L: EuroQol 5 dimensions; HADS: Hospital Anxiety and Depression Scale; MFI-20: Multidimensional Fatigue Inventory; PCFS: post-COVID functional status; SD: standard deviation; SG-PALS: Saltin Grimby physical activity level scale; VAS: visual analog scale.

## Factors associated with aspects of functioning 1 year after hospitalization due to COVID-19

**Figure 3.**
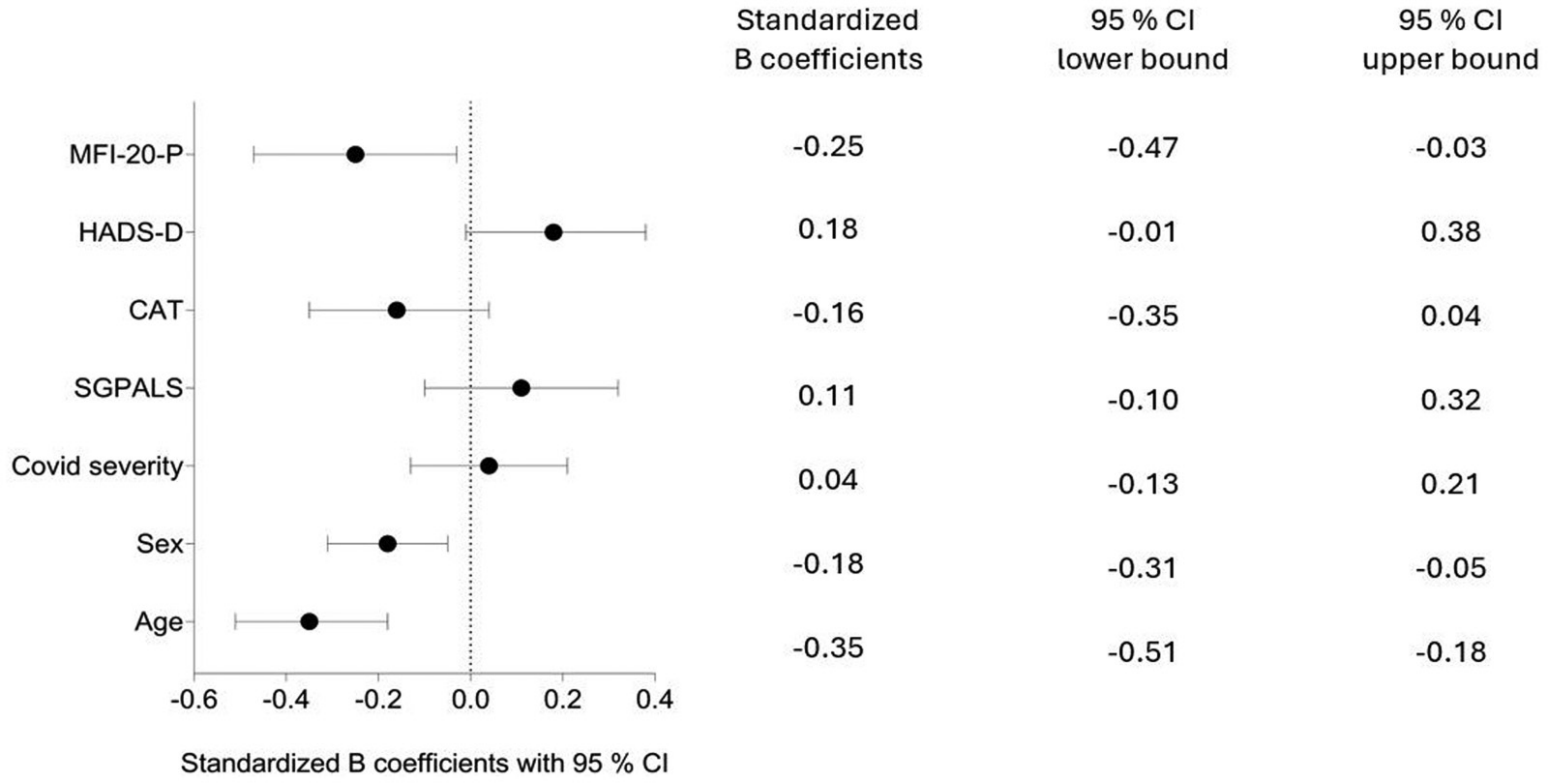
Associations of factors 3 months after COVID-19 and walking distance 1 year after hospitalization due to COVID-19 according to multivariate linear regression.

Older age (*p* < 0.001), being female (*p* = 0.008), and having higher levels of physical fatigue 3 months after discharge (*p* = 0.027), were significantly associated with lower functioning 1 year after COVID-19 (Figure 3).

## Discussion

The present study highlights factors at 3 months that warrant special attention due to their importance for outcomes at 1 year. The study showed that age, sex, and fatigue are associated with functioning after COVID-19. However, previous severity of the COVID infection was not significantly associated with functioning. Similarly, symptoms of depression, level of physical activity after COVID-19 and respiratory symptoms, were not significantly associated with functioning 1 year after hospitalization. These findings suggest that interventions targeting fatigue and considering demographic factors such as age and sex may be critical for improving long-term recovery, particularly for individuals previously hospitalized due to COVID-19. These insights are valuable for healthcare systems globally, as they address the long-term effects of COVID-19 and can help inform strategies for rehabilitation and posthospital care.

In this study, discrepancy between self-reported respiratory difficulties and clinical measurements, such as spirometry may be due to several factors. Screening respiratory functions with spirometry can indicate impaired lung function but limits the insight into respiratory functions of the small peripheral airways.^22^ In the present study, more participants were below the normative values for forced expiratory volume in 1 second than for forced vital capacity. The impairments in forced expiratory volume in 1 second indicate reduced function of the small peripheral airways or obstructive airways.^22^ In addition, 15% of participants had exercise-induced desaturation after the *6-minute walk test*. These problems could be due to several factors, such as respiratory dysfunction or endothelial dysfunction, which have been described after COVID-19.^32^ Respiratory functions may be limited by altered respiratory patterns,^33^ abnormal gas exchange, and autonomic dysfunction.^3,22,32^ The ability to swiftly move air in and out of the lungs is an essential function for daily activities, and any limitation in respiratory function will cause breathlessness and limit activities and exercise tolerance.^22^

In the present study, the walking distance on the *6-minute walk test* was set as a proxy for functioning 1 year after COVID-19. An individual's ability to walk a distance with ease is an important factor of quality of life because it is highly associated with the ability to perform everyday activities.^34^ The participants walked 441 m on average, which is similar to what was described in another Swedish study of nonhospitalized individuals recovering from COVID-19.^35^ Walking distance is influenced by several factors, such as age, fitness, height, and ethnicity, and normal ranges in healthy adults are estimated to vary between 400 and 700 m,^34^ with < 300 m being linked to frailty and mortality.^36^ In this study, 11% of participants walked a distance  < 300 m, indicating that the results in the present study cohort are at the lower limits of normal ranges. The mean number of chair rises was 11.8 (SD 5.3), which can be compared to normative values for individuals of a similar age (mean 67 years), who range between 11 and 18 chair rises.^25^ However, direct comparisons are challenging, as reference values primarily represent data from older adults, making generalization of the leg strength difficult. When assessing strength in the upper extremities the observed percentage of predicted value was within normative or above.^27^ In summary, it is possible that individuals with the previously discussed respiratory difficulties after COVID-19 have the strength to manage their activities but do so very close to their maximal level of exertion.

In the present study, higher levels of physical fatigue 3 months after COVID-19 were associated with lower levels of functioning 1 year after hospitalization. Although high variation was observed in the median values, higher levels of physical fatigue were noted compared to the normative values from the Swedish population.^8^ Physical activity can be seen as essential when managing fatigue.^37,38^ Post-COVID with respiratory symptoms may limit physical activity and rehabilitation, with prior findings indicating that individuals recovering from COVID-19 are less physically active.^9,17,35^ In this cohort, 40% of participants reported some degree of mobility problems. Physical activity and exercise increase the respiratory breathing effort and may lead to muscle fatigue and increase the sensation of dyspnea, especially in individuals with respiratory disease.^39^ Taking this into consideration, it is possible that the physical fatigue that this patient group presents can be explained, in part, by the high energy consumption spent on respiratory muscle work while engaging in physical activity and activities of everyday life.

A reduction in functional capacity can be expected with age and is also influenced by sex.^40^ The independent variables age and sex could overshadow other variables in the analysis, explaining the moderate *r*^2^ of 0.31. Our results indicate that older age, female sex, and greater levels of fatigue are indicators of lower levels of functioning 1 year after COVID-19, whereas other factors, such as COVID severity did not significantly impact the results.

While these findings provide insight in the return of function after COVID-19, it is important to acknowledge the strengths and limitations with this study. To the best of our knowledge, this is the largest multicenter observational study covering functional outcomes after hospitalization due to COVID-19 in Sweden during the first and second waves of the pandemic. As of that these results could be generalizable to individuals in Sweden who were hospitalized with COVID-19, within this timeframe. Two patient partners were involved throughout the project, providing valuable insights into matters of importance for individuals treated for COVID-19. Although the study aimed for consecutive inclusion, the potential for selection bias due to the pandemic and the increased workload in the clinics cannot be dismissed. This may limit the generalizability of the findings to broader populations or settings. Furthermore, the absence of a control group is a limitation of the present study, restricting the understanding of general changes. However, including additional participants was not feasible due to the constraints of the clinical setting. Some participants did not have a weight registered when screening the medical charts, leading to limited data analysis, such as reference equations for normative values regarding walking distance.

One year after hospitalization due to COVID-19 some individuals still suffer from functional impairments. This study shows that individuals with adequate lung function on spirometry and adequate walking distance, may still report high levels of respiratory difficulties and fatigue, which may indicate that their functional performance is close to their maximal capacity. These results indicate that in order to detect functional impairments after hospitalization due to COVID-19 clinicians may need to assess individuals more in-depth, to identify the need of and provide adequate rehabilitation.

**Clinical messages**

- One year after COVID-19, previously infected individuals might still suffer from functional impairments which highlights the need for in-depth assessments to identify rehabilitation needs.
- One year after COVID-19, individuals who were previously hospitalized may present acceptable results in clinical testing but be functioning on a level close to maximal exertion.
- Individuals who are feeling physically fatigued may have lower levels of functioning 1 year after COVID-19. Extra support from physical therapy and accessible rehabilitation could be beneficial for these patients.
- The findings suggest that targeting fatigue and considering demographic factors such as age and sex may be critical for improving long-term recovery after hospital care.

## Supplemental Material

sj-pdf-1-cre-10.1177_02692155241311852 - Supplemental material for Factors associated with aspects of functioning one year after hospitalization due to COVID-19 Supplemental material, sj-pdf-1-cre-10.1177_02692155241311852 for Factors associated with aspects of functioning one year after hospitalization due to COVID-19 by Alexandra C. Larsson, Annie Palstam, Linda Ashman Kröönström, Katharina S. Sunnerhagen and Hanna C. Persson in Clinical Rehabilitation
